# CD44 Can Compensate for IgSF11 Deficiency by Associating with the Scaffold Protein PSD-95 during Osteoclast Differentiation

**DOI:** 10.3390/ijms21072646

**Published:** 2020-04-10

**Authors:** Hyunsoo Kim, Noriko Takegahara, Matthew C. Walsh, Yongwon Choi

**Affiliations:** Department of Pathology and Laboratory Medicine, University of Pennsylvania Perelman School of Medicine, Philadelphia, PA 19104, USA; hyunsoo3@pennmedicine.upenn.edu (H.K.); tnoriko@pennmedicine.upenn.edu (N.T.); mcw@pennmedicine.upenn.edu (M.C.W.)

**Keywords:** Osteoclast, CD44, IgSF11, PSD-95, differentiation

## Abstract

Differentiation of osteoclasts, which are specialized multinucleated macrophages capable of bone resorption, is driven primarily by receptor activator of NF-κB ligand (RANKL). Additional signaling from cell surface receptors, such as cell adhesion molecules (CAMs), is also required for osteoclast maturation. Previously, we have demonstrated that immunoglobulin superfamily 11 (IgSF11), a member of the immunoglobulin-CAM (IgCAM) family, plays an important role in osteoclast differentiation through association with the scaffold protein postsynaptic density protein 95 (PSD-95). Here, we demonstrate that the osteoclast-expressed CAM CD44 can compensate for IgSF11 deficiency when cell–cell interaction conditions are suboptimal by associating with PSD-95. Impaired osteoclast differentiation in IgSF11-deficient (IgSF11^−/−^) cultures was rescued by antibody-mediated stimulation of CD44 or by treatment with low-molecular-weight hyaluronan (LMW-HA), a CD44 ligand. Biochemical analysis revealed that PSD-95, which is required for osteoclast differentiation, associates with CD44 in osteoclasts regardless of the presence or absence of IgSF11. RNAi-mediated knockdown of PSD-95 abrogated the effects of either CD44 stimulation or LMW-HA treatment on osteoclast differentiation, suggesting that CD44, similar to IgSF11, is functionally associated with PSD-95 during osteoclast differentiation. Taken together, these results reveal that CD44 can compensate for IgSF11 deficiency in osteoclasts through association with PSD-95.

## 1. Introduction

Skeletal bone is maintained via continuous bone formation and destruction mediated by osteoblasts and osteoclasts [1,2]. Functional imbalance between osteoblasts and osteoclasts results in various skeletal disorders. Osteoclasts are vital for maintaining a healthy skeleton, but excessive activity or developmental and functional defects of osteoclasts are associated with numerous pathophysiological processes in bone [3]. Therefore, understanding the processes that control osteoclast biology is necessary to provide a molecular basis for designing therapeutic strategies for these diseases.

Osteoclasts are specialized multinucleated giant cells capable of bone resorption [4,5] and are differentiated from myeloid lineage progenitors by stimulation with the osteoclast differentiation factor RANKL, which is mainly provided by osteoblasts and osteocytes [6,7,8]. Upon stimulation with RANKL, myeloid precursors undergo incomplete cytokinesis and/or cell fusion to become multinucleated [9], a hallmark of osteoclast maturation [4,5]. Cell–cell interactions mediated by cell surface receptors and/or cell adhesion molecules (CAMs) are required not only to mediate cell fusion but also to provide co-stimulatory signaling necessary for proper osteoclast differentiation and maturation [10,11]. Therefore, cell–cell interaction is critical for the differentiation and maturation of osteoclasts, and numerous osteoclast-expressed CAMs have been identified. IgSF11 is a member of the coxsackievirus and adenovirus receptor (CAR) group of the CTX (the cortical thymocyte marker in Xenopus) family of transmembrane immunoglobulin-like CAMs [12,13]. IgSF11 has an extracellular domain with a membrane-distal V-type domain and a membrane-proximal C2-type domain, a transmembrane domain, and a cytoplasmic domain with PDZ binding motif at C-terminal. We recently demonstrated a critical role for IgSF11 in osteoclast differentiation, with IgSF11 deficiency resulting in impaired osteoclast differentiation [14]. We found that IgSF11 functions through homophilic interactions. Additionally, IgSF11 associates with the scaffold protein PSD-95 through the intracellular C-terminal region of IgSF11. PSD-95 is a specialized scaffold protein with multiple protein interaction domains and forms the backbone of an extensive postsynaptic protein complex that organizes receptors and signal transduction molecules at the synaptic contact zone [15]. Therefore, IgSF11 appears to function as both a cell surface receptor and signal transduction molecule-containing protein complex required for osteoclast differentiation.

CD44, a type I transmembrane glycoprotein that acts as a CAM [16,17,18], is ubiquitously expressed throughout the body. The primary domains of CD44 are the extracellular domain, the transmembrane domain, and the cytoplasmic domain [17]. The extracellular domain interacts with the external microenvironment and links the extracellular matrix (ECM) components and the cell surface. This link directs intracellular signaling as well as organization and modification of the ECM. CD44 was originally identified as a receptor for hyaluronic acid (HA) which is a long, unbranched high-molecular-weight (~10^7^ Da) polymer composed of repeating glucuronic acid and N-acetyl glucosamine disaccharide units [17,19,20]. The molecular weight of HA may be decreased due to free radical depolymerization of the HA chain and/or abnormal biosynthesis by the synovium, resulting in generation of low-molecular-weight hyaluronan (LMW-HA) [21,22]. Later, numerous ECM components including collagen, laminin, and fibronectin were identified as able to bind CD44 [18,23,24,25]. In addition, a number of glycosaminoglycans, including osteopontin, have been reported to bind to CD44 [26]. The interaction of CD44 with its ligands has been shown to regulate cell adhesion, cell motility, matrix degradation, cell proliferation and survival. Additionally, CD44 is activated by matrix metalloproteinases such as membrane type 1 matrix metalloprotease [17,27], and proteolytically cleaved by γ-secretase to produce an intracytoplasmic domain called CD44-ICD which activates the expression of many genes [28]. In osteoclasts, CD44 has been linked to cell fusion [29,30,31,32]. However, the specific functions and underlying mechanisms of CD44 in osteoclasts still remain largely unknown.

In this study, we identified a compensatory relationship between IgSF11 and CD44 in regulation of osteoclast differentiation. Using an in-vitro culture system, we revealed that under conditions of suboptimal cell–cell interaction CD44 can compensate IgSF11 deficiency in osteoclasts. Additionally, we demonstrated that PSD-95 associates with CD44 in osteoclasts, and is required for CD44 function. Our findings provide evidence of a role for CD44 in regulation of osteoclast differentiation, and further suggest the importance of CAMs in osteoclast differentiation through association with PSD-95.

## 2. Results

### 2.1. Impaired Osteoclast Differentiation in IgSF11-Deficient Culture Is Rescued by Increased Cell Density

We have previously reported that ablation of the gene IgSF11 in mice results in increased bone mass, not due to altered osteoblast activity, but rather because of impaired osteoclast differentiation [14]. Indeed, when bone marrow monocytes (BMMs) from wild-type (IgSF11^+/+^) and IgSF11-deficient (IgSF11^−/−^) mice were cultured with M-CSF + RANKL to induce osteoclasts, IgSF11^−/−^ cultures showed decreased numbers of tartrate-resistant acidic phosphatase-positive (TRAP^+^) multinuclear cells (i.e., mature osteoclasts), consistent with our previous findings (Figure S1) [14]. However, given the importance of cell–cell interactions to osteoclast differentiation and maturation, we hypothesized that high cell culture density would increase the probability of cell–cell interactions that might be mediated by alternative cell surface molecules in the absence of IgSF11 homophilic interactions. Indeed, we found that increased cell density increased formation of mature osteoclasts from IgSF11^−/−^ cultures to a comparable level seen in IgSF11^+/+^ cultures (Figure S1A). We then performed temporal osteoclast differentiation assays in low-cell-density and high-cell-density cultures (Supplemental Figure S1B). Consistent with our previous report [14], impaired osteoclast differentiation in IgSF11^−/−^ cultures was observed from day 2, and the differences became bigger on day 3 in low-cell-density cultures. On the other hand, IgSF11^+/+^ and IgSF11^−/−^ cells cultures at a high cell density showed no differences in terms of osteoclast formation.

### 2.2. CD44 Stimulation Rescues Impaired IgSF11-Deficient Osteoclast Differentiation

**Figure 1:**
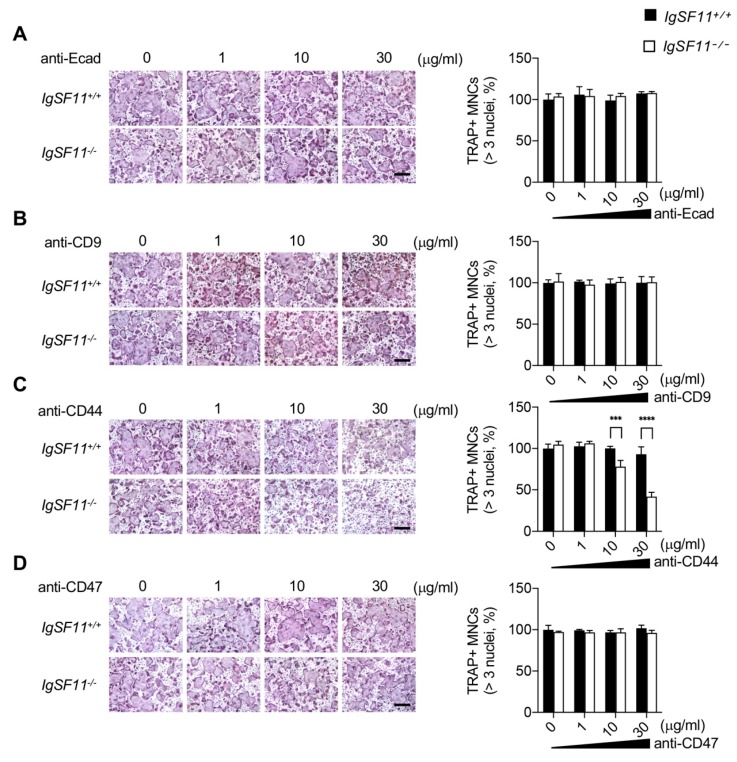
Blocking CD44 inhibits osteoclast differentiation of IgSF11-deficient cells in high-cell-density cultures. High-cell-density cultures of IgSF11^+/+^ and IgSF11^−/−^ BMMs were treated with the indicated doses of soluble antibodies (**A**) anti-E-cadherin, (**B**) anti-CD9, (**C**) anti-CD44, and (**D**) anti-CD47, and cultured with M-CSF + RANKL for three days to induce osteoclast differentiation. On day three, cells were fixed and stained with TRAP (left). TRAP^+^ multinucleated cells (three nuclei or more per cell) were counted and the frequency of TRAP^+^ multinucleated cells is shown (right). Scale bars represent 100 μm. Data are shown as the mean ± S.D. *** *p* < 0.001, **** *p* < 0.0001.

We sought to identify the molecule(s) responsible for compensation of IgSF11 deficiency under high-cell-density conditions, and performed blocking experiments using antibodies against cell surface molecules known to be involved in osteoclast differentiation/multinucleation, including E-cadherin, CD9, CD44, and CD47, [32,33,34,35]. High-cell-density cultures of IgSF11^+/+^ or IgSF11^−/−^ BMMs were treated with antibodies against these molecules and cultured with M-CSF + RANKL. Treatment with anti-CD44 but not with anti-E-cadherin, anti-CD9, or anti-CD47 inhibited formation of TRAP^+^ multinucleated cells in high-cell-density IgSF11^−/−^ cultures in a dose-dependent manner (Figure 1). However, anti-CD44 treatment did not result in inhibitory effects on formation of TRAP^+^ multinucleated cells in high-cell-density IgSF11^+/+^ cultures (Figure 1). These results suggested that CD44 can compensate for the effects of IgSF11 deficiency on osteoclast formation in high-cell-density cultures, and also implied a potential role for CD44 that may be masked by the presence of IgSF11 under high-cell-density culture conditions.

When IgSF11^+/+^ and IgSF11^−/−^ cells cultured at a low cell density (i.e., a cell density at which IgSF11 deficiency results in impaired osteoclast differentiation) were treated with anti-CD44, dramatic reductions in TRAP^+^ multinucleated cell formation were observed in both IgSF11^+/+^ and IgSF11^−/−^ cultures (Figure S2). These results supported the idea that cell density affects CAM-mediated cell–cell interactions and regulation of osteoclast differentiation. Together, these results suggest an interrelationship between IgSF11 and CD44 in the context of osteoclast differentiation.

**Figure 2:**
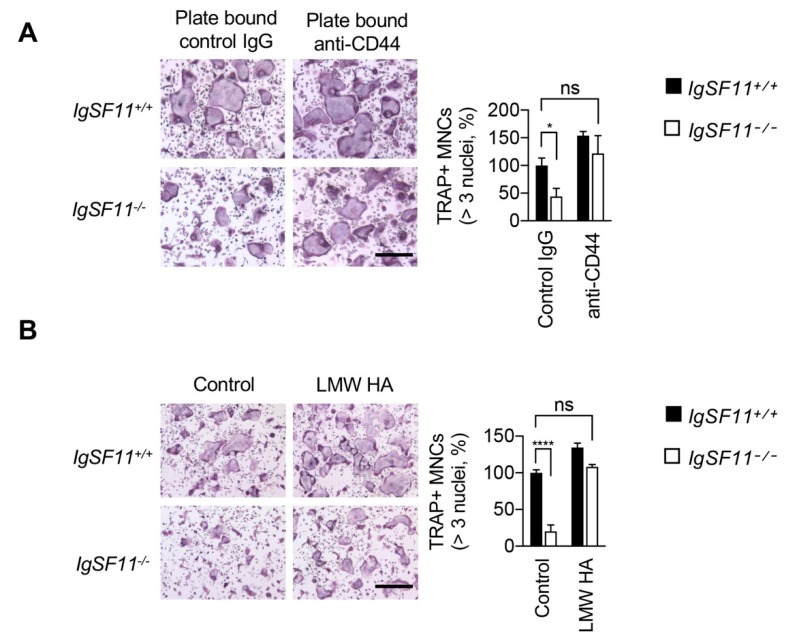
Stimulation of CD44 rescues impaired IgSF11-deficient osteoclast differentiation. IgSF11^+/+^ and IgSF11^−/−^ BMMs were (**A**) seeded on plate-bound control IgG or anti-CD44 at low cell density or (**B**) treated with LMW HA at low cell density, and cultured with M-CSF + RANKL for three days to induce osteoclast differentiation. On day three, cells were fixed and stained with TRAP (left). TRAP^+^ multinucleated cells (three nuclei or more per cell) were counted and the frequency of TRAP^+^ multinucleated cells is shown (right). Scale bars represent 100 μm. Data are shown as the mean ± S.D. * *p* < 0.05, **** *p* < 0.0001, ns; not significant.

To further investigate the interrelationship between CD44 and IgSF11 in osteoclasts, we examined the effect of CD44 stimulation on IgSF11^−/−^ cells. We employed plate-bound (rather than blocking/neutralizing soluble) anti-CD44, since immobilized antibody can cross-link and stimulate CD44-dependent signaling. IgSF11^+/+^ and IgSF11^−/−^ BMMs were cultured on plate-bound anti-CD44 at low cell density, and cultured with M-CSF + RANKL to induce differentiation to osteoclasts. Stimulation with anti-CD44 rescued impaired TRAP^+^ multinucleated cell formation in IgSF11^−/−^ cultures (Figure 2A). CD44 has multiple ligands, including low-molecular-weight hyaluronan (LMW-HA), which has been shown to stimulate osteoclast formation [21]. We stimulated low-cell-density IgSF11^+/+^ and IgSF11^−/−^ cultures with LMW-HA and found that LMW-HA rescued TRAP^+^ multinucleated cell formation in IgSF11^−/−^ cultures (Figure 2B). These results showed that CD44 stimulation could rescue the effects of IgSF11 deficiency in osteoclasts, and suggested that CD44 can act in an IgSF11-independent manner.

**Figure 3:**
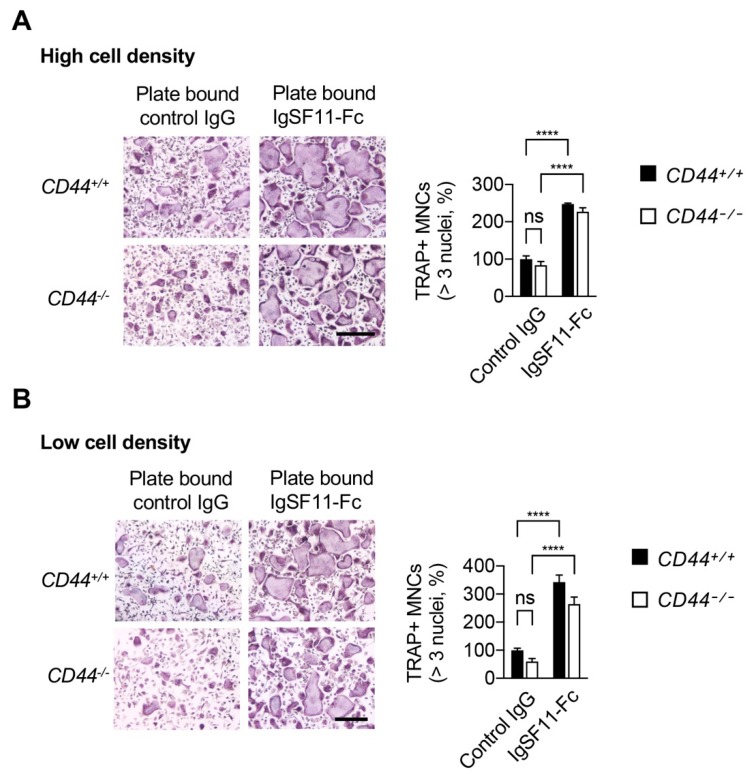
Stimulation of IgSF11 exerts a pro-osteoclastogenic effect in a CD44-independent manner. CD44^+/+^ and CD44^−/−^ BMMs were seeded on plate-bound control IgG or IgSF11-Fc at (**A**) high cell density, or (**B**) low cell density, and cultured with M-CSF + RANKL for three days to induce osteoclast differentiation. On day three, cells were fixed and stained with TRAP (left). TRAP^+^ multinucleated cells (three nuclei or more per cell) were counted and the frequency of TRAP^+^ multinucleated cells is shown (right). Scale bars represent 100 μm. Data are shown as the mean ± S.D. **** *p* < 0.0001, ns; not significant.

Next, we examined the effect of IgSF11 stimulation on CD44-deficient (CD44^−/−^) cells. As we reported previously, IgSF11 engages in homophilic interactions, and adding recombinant soluble IgSF11-Fc protein that consists of the extracellular region of IgSF11 fused to the human IgG Fc region blocked osteoclast differentiation in a dose-dependent manner (Figure S3) [14]. We therefore employed plate-bound recombinant IgSF11-Fc protein to instead stimulate IgSF11 (Figure 3). CD44^+/+^ and CD44^−/−^ BMMs were cultured at high cell density (Figure 3A) or low cell density (Figure 3B) on plate-bound IgSF11-Fc with M-CSF + RANKL. Although CD44 deficiency did not result in significant reduction of TRAP^+^ multinucleated cell formation at either cell density (Figure 3A,B), stimulation with IgSF11-Fc dramatically enhanced formation of TRAP^+^ multinucleated cells in both CD44^+/+^ and CD44^−/−^ cultures (Figure 3). These results suggested that direct stimulation of IgSF11 is capable of exerting a pro-osteoclastogenic effect in a CD44-independent manner, and that CD44 deficiency alone does not negatively impact in-vitro osteoclast differentiation.

### 2.3. PSD-95 Is Required for CD44-Mediated Osteoclast Differentiation

**Figure 4:**
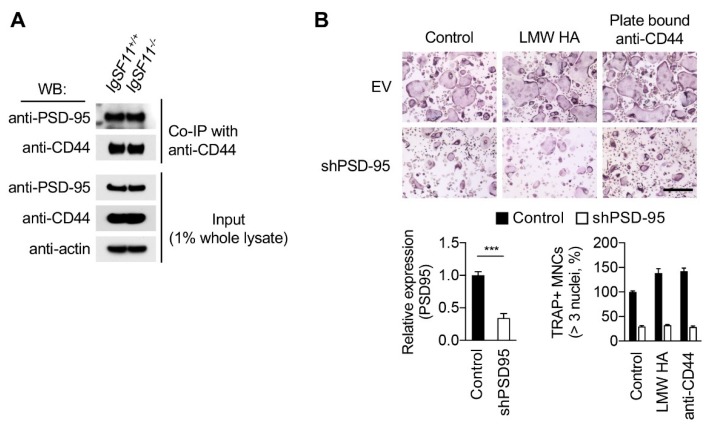
PSD-95 is required for CD44-mediated osteoclast differentiation. (**A**) Coimmunoprecipitation (Co-IP) assay. IgSF11^+/+^ and IgSF11^−/−^ pre-osteoclasts were lysed and the lysates were immunoprecipitated with anti-CD44 antibody. Western blotting (WB) was performed with the indicated antibodies. Input shows amount of proteins in the lysates. (**B**) Effect of knockdown of PSD-95 on CD44 stimulation-induced osteoclast differentiation. Wild-type BMMs retrovirally transduced with the indicated shRNAs (EV, empty vector control) were cultured with M-CSF + RANKL in the presence of the indicated stimuli (LMW HA and/or plate bound anti-CD44) for three days. On day three, cells were fixed and stained for TRAP (top). Relative expression of PSD-95 was determined by Q-PCR (bottom left). TRAP^+^ multinucleated cells (three nuclei or more per cell) were counted and the frequency of TRAP^+^ multinucleated cells is shown (bottom right). Scale bars represent 100 μm. Data are shown as the mean ± S.D. *** *p* < 0.001.

We sought to further investigate the molecular mechanism underlying the interrelationship between IgSF11 and CD44 in osteoclasts. Given the requirement for association between IgSF11 and PSD-95 during osteoclast differentiation [14] and the capacity of CD44 to compensate for IgSF11 deficiency, we hypothesized that CD44 may functionally associate with PSD-95 during osteoclast differentiation. To examine the association of CD44 with PSD-95, BMMs were cultured with M-CSF + RANKL for two days to induce pre-osteoclasts, and the lysates were used for coimmunoprecipitation. Association of PSD-95 with CD44 was detected in wild-type cultures (Figure 4A), and also detected in IgSF11^−/−^ cultures (Figure 4A), suggesting that CD44 associates with PSD-95 independently of IgSF11. To further examine the involvement of PSD-95 in CD44-mediated regulation of osteoclast differentiation, BMMs were retrovirally transduced with shRNA encoding PSD-95 to knock down expression of PSD-95, followed by stimulation of CD44. As previously reported, RNAi-mediated knockdown of PSD-95 inhibits osteoclast differentiation (Figure 4B) [14]. Neither stimulation with plate-bound anti-CD44 nor treatment with LMW-HA showed stimulatory effects on osteoclast formation in PSD-95 knockdown cultures (Figure 4B). These results suggested that PSD-95 is required for CD44-mediated osteoclast differentiation.

## 3. Discussion

RANKL stimulation drives the commitment of osteoclast precursors to become large multinucleated mature osteoclasts. In addition to RANKL stimulation, cell–cell adhesion through cell surface receptors is required not only to mediate cell fusion but also to provide co-stimulatory signaling necessary for proper osteoclast differentiation and maturation. CAMs in particular are required to establish cell–cell contacts and to mediate intracellular signaling for optimal activation and/or differentiation of osteoclasts. In this study, we revealed an interrelationship between the osteoclast-expressed CAMs CD44 and IgSF11, which regulates osteoclast differentiation. Further, as in the case of IgSF11-mediated osteoclast differentiation, we identified a requirement for PSD-95 in the context of CD44-mediated regulation of osteoclast differentiation. Together, our findings reveal new critical roles for CAMs during osteoclast differentiation.

In this study, we performed temporal osteoclast differentiation assays to show a compensatory effect on IgSF11 deficiency by increasing cell density, which was observed from an early (day two) stage (Supplemental Figure S1B), emphasizing the importance of CAM-mediated cell–cell interactions during osteoclast differentiation. We did not address the effect of long-term culture (more than 3 days) since fully mature osteoclasts, which are generated by 3 days of culture, will be highly susceptible to cell death after 3 days. However, we cannot exclude possible effects of long-term culture on CAM-mediated regulation of osteoclast differentiation. Further studies will be needed to address this issue.

CD44 has been reported to be involved in osteoclast multinucleation [29,30,31,32]. However, the precise function and underlying mechanism have remained undetermined [36,37,38]. Using antibody against CD44, we showed here that blocking CD44 inhibited osteoclast formation, while stimulation of CD44 rescued impaired osteoclast differentiation in IgSF11-deficient cultures. These results revealed a positive regulatory function for CD44 in osteoclast differentiation. Of note, we also showed that CD44 deficiency alone does not impact in-vitro osteoclast formation, and that stimulation of IgSF11 dramatically enhances osteoclast formation independently of CD44 (Figure 3). Given that IgSF11 deficiency results in increased bone mass [14] while CD44 deficiency causes no obvious developmental defects [39,40], our observations suggest that IgSF11-mediated signaling might be strong enough to support osteoclast differentiation under normal physiological conditions, and that CD44 might exert complementary functions during osteoclast differentiation. CD44 has multiple ligands, including extracellular matrixes. Although we showed here that LMW-HA stimulates CD44, we cannot exclude the possibility that other extracellular matrixes/molecules contribute to CD44 stimulation, compensating for a lack of IgSF11 and supporting osteoclast differentiation. Further studies will be required to fully understand how and when CD44 is stimulated during physiologic osteoclast differentiation.

We sought to identify the molecular mechanisms by which CD44 compensates IgSF11 in osteoclasts, and identified association of CD44 with PSD-95, a molecule that also associates with IgSF11 by biochemical analysis. Protein expression of PSD-95 and CD44 was comparable between IgSF11^+/+^ and IgSF11^−/−^, and association of CD44 and PSD-95 was observed in IgSF11^−/−^ cultures (Figure 4A), suggesting that expression of and interaction between CD44 and PSD-95 in osteoclasts are not perturbed by IgSF11 deficiency. Additionally, using RNAi experiments in which Q-PCR analysis revealed 65.6 ± 7% knockdown of PSD-95 expression, we found stimulation of CD44 failed to rescue the impaired osteoclast differentiation (Figure 4B), suggesting critical involvement of PSD-95 downstream of CD44 signaling in the context of osteoclast differentiation. Given the function and requirement for PSD-95 in osteoclasts [14], it is plausible that PSD-95 acts as a signaling platform that regulates signal transduction via CAMs at the cell–cell contact site during osteoclast differentiation. In addition to our previous finding that IgSF11 associates with PSD-95 through its 75 C-terminal amino acids in osteoclasts [14], C-terminal PDZ-binding motif of IgSF11 has been demonstrated to be involved in interaction with PSD-95 [41]. Given that PDZ domains in PSD-95 typically bind C-terminal tails of four to five residues from other proteins [42], IgSF11 C-terminal PDZ binding motif might be essential for association with PSD-95 in osteoclasts. However, the structural requirements of CD44 for interaction with PSD-95 remains to be determined. Future studies will further clarify how CAMs, together with the PSD-95 protein complex, regulates osteoclast differentiation.

Taken together, we provide evidence here of an interrelationship between IgSF11 and CD44, and further, a critical role for PSD-95 as a lynchpin for a protein complex that organizes CAMs and signal transduction molecules within developing osteoclasts. Our results show the importance of cell–cell interactions mediated by CAMs to support osteoclast differentiation. Finally, it is likely that continued identification and characterization of additional regulators of osteoclast differentiation may further aid in the development of therapeutic strategies for the treatment of skeletal diseases.

## 4. Materials and Methods

### 4.1. Mice

IgSF11^−/−^ mice were generated as previously described [14]. CD44^−/−^ mice were purchased from the Jackson Laboratory. All mice were maintained and used in accordance with guidelines approved by the Institutional Animal Care and Use Committee (IACUC) at the University of Pennsylvania (IACUC #804178, approved on 27 February 2018).

### 4.2. In Vitro Osteoclast Differentiation and Tartrate-Resistant Acid Phosphatase (TRAP) Staining

Wild-type, IgSF11^−/−^, and CD44^−/−^ mouse bone marrow derived monocytes (BMMs) and osteoclasts were prepared as described previously [43]. In brief, whole bone marrow was extracted from the femurs and tibias of mice and incubated in 100 mm petri dishes in α-MEM medium containing 10% fetal bovine serum and M-CSF (5 ng/mL) overnight. Non-adherent cells were collected and cultured for 3 days with M-CSF (30 ng/mL) to generate BMMs. For osteoclast differentiation, BMMs were plated at 5 × 10^3^ per well (low cell density) or 3 × 10^4^ per well (high cell density) in 96-well cell culture plates and cultured with M-CSF (60 ng/mL) and RANKL (150 ng/mL) for 3 days. For the blocking experiment, soluble form antibodies (in high-cell-density culture: 1, 10 and 30 μg/mL, and in low-cell-density culture: 0.3, 1, 3, and 9 μg/mL) or recombinant protein (0.33, 1.3, 5, and 10 μg/mL) were added in the culture. For plate-bound stimulation assay, antibody or recombinant protein were coated on 96-well plates with coating buffer (50 mM Tris-HCl, pH 9.5) for overnight at 4 °C. Coated plates were washed with culture medium 3 times before seeding cells. Osteoclasts were stained using the acid phosphatase leukocyte kit (Tartrate-Resistant Acid Phosphatase Kit (387A-1KT, Sigma-Aldrich, St.Louis, MO, USA)) following the manufacturer’s instructions. Anti-E-cadherin (DECMA-1: #16-3249-85, Thermoscientific, Fremont, CA, USA), anti-CD9 (KMC8: #553758, BD Pharmingen, San jose, CA, USA), anti-CD44 (IM7: #103046, Biolegend, San Diego, CA, USA), and anti-CD47 (miap301: #16-0471-85, eBioscience, San Diego, CA, USA) were purchased. Recombinant IgSF11-Fc was prepared as described previously [14]. LMW-HA was purchased from R&D system (Minneapolis, MN, USA) (#GLR001) and used at 90 μg/mL.

### 4.3. Co-Immunoprecipitation (Co-IP) and Western Blot

Cell cultures were washed with ice-cold phosphate-buffered saline (PBS) and lysed with ice-cold radio immunoprecipitation (RIPA) lysis buffer (20 mM Tris-HCl, pH 7.5, 150 mM NaCl, 1% NP-40, 0.5% sodium deoxycholate, 1 mM EDTA, 0.1% SDS, protease and phosphatase inhibitor cocktail (Roche, Lewes, UK)). The lysates were centrifuged to remove debris, and protein concentrations were determined using the Bradford assay. Equal amounts of lysates (20–80 μg of protein) were pull-downed using protein G beads conjugated with antibodies or control IgG for overnight. The beads were washed and fractionated by SDS-polyacrylamide gel electrophoresis (SDS-PAGE) on 4–12% gradient gels and transferred onto polyvinyl difluoride (PVDF) membranes. Western blotting was performed with the following antibodies: anti-PSD-95: K28/43 (Biolegend), anti-CD44: 37259 (Cell Signaling Technology, Danvers, MA, USA), and anti-Actin: sc-47778 (Santacruz Biotechnology, Santa Cruz, CA, USA).

### 4.4. Retrovirus Preparation and Transduction

To prepare retroviral particles, Plat-E packaging cells were plated on 100 mm culture dishes and transfected with pSuper vectors encoding shRNA targeting PSD-95 (sh-PSD-95-1) [14] and empty vector using PEImax (Polysciences, Warrington, PA, USA). After 3 days, medium containing each retrovirus was harvested and passed through a syringe filter (0.45 μm pore diameter). BMMs were transduced with retroviruses for 16 h with hexadimethrine bromide (8 μg/mL) in the presence of M-CSF (60 ng/mL). After washing with fresh medium, infected cells were selected by culturing for 2 days in the presence of puromycin (2 μg/mL) and M-CSF (60 ng/mL). Puromycin-resistant BMMs were used for the experiments.

### 4.5. Reverse Transcription and Real-Time PCR (Q-PCR)

Total RNA was extracted from cells using TRIzol reagent (Invitrogen, Carlsbad, CA, USA). The RNA quality was determined by detecting its absorbance ratio at 260:280 nm wavelength using a NanoDrop Spectrophotometer (ThermoScientific, Fremont, CA, USA), and confirmed that the ratio value was 2.0 and over. One to five μg of total RNA was reverse transcribed using random hexamer primers and SuperScript III reverse transcriptase (Invitrogen, Carlsbad, CA, USA). cDNA corresponding to 10 ng of total RNA was analyzed by Q-PCR using a QuantStudio3 (Applied Biosystems, San Francisco, CA, USA) and the following specific TaqMan probes: PSD-95 (Mm00492193_m1), and 18S (Hs99999901_s1). The ddCT method of relative quantification was used to determine the fold change in expression.

### 4.6. Statistical Analysis

All experiments were analyzed using one-way ANOVA or two-tailed paired Students *t*-test with Prism 8.3 (GraphPad Software, San Diego, CA, USA). *p* < 0.05 was considered statistically significant.

## 5. Conclusions

The main finding of this study is that an osteoclast-expressed CAM, CD44, can compensate for IgSF11 deficiency by associating with PSD-95 during osteoclast differentiation. Our findings reveal a compensatory relationship between IgSF11 and CD44 to regulate osteoclast differentiation through association with PSD-95. These findings highlight new critical roles for CAMs during osteoclast differentiation. A limitation of this study is the lack of characterization of the relationship between IgSF11 and CD44 in osteoclasts in vivo due to difficulties of changing/manipulating cell densities under physiological circumstances.

## Supplementary Materials

Supplementary Materials can be found at https://www.mdpi.com/1422-0067/21/7/2646/s1. Figure S1. Increased cell density rescues impaired in vitro IgSF11-deficient osteoclast differentiation. (**A**) IgSF11^+/+^ and IgSF11^−/−^ BMMs were cultured at low cell density (5 × 10^3^ cells/96-well plate well) or high cell density (3 × 10^4^ cells/96-well plate well) with M-CSF + RANKL for three days to induce osteoclast differentiation. On day three, cells were fixed and stained with TRAP (left). TRAP^+^ multinucleated cells (three nuclei or more per cell) were counted and the frequency of TRAP^+^ multinucleated cells is shown (right). Scale bars represent 100 μm. (**B**) IgSF11^+/+^ and IgSF11^−/−^ BMMs were cultured at low cell density (5 × 10^3^ cells/96-well plate well) or high cell density (3 × 10^4^ cells/96-well plate well) with M-CSF + RANKL for three days to induce osteoclast differentiation. Cells were fixed day by day (from day zero to day three) and stained with TRAP. TRAP^+^ multinucleated cells (three nuclei or more per cell) were counted and the frequency of TRAP^+^ multinucleated cells is shown. Data are shown as the mean ± S.D. *** *p* < 0.001, **** *p* < 0.0001, ns; not significant. Figure S2. Effect of blocking antibodies against osteoclast differentiation/multinucleation-related molecules. Low-cell-density cultures of IgSF11^+/+^ and IgSF11^−/−^ BMMs were treated with the indicated doses of soluble antibodies (**A**) anti-E-cadherin, (**B**) anti-CD9, (**C**) anti-CD44, and (**D**) anti-CD47, and cultured with M-CSF + RANKL for three days to induce osteoclast differentiation. On day three, cells were fixed and stained with TRAP (left). TRAP^+^ multinucleated cells (three nuclei or more per cell) were counted and the frequency of TRAP^+^ multinucleated cells is shown (right). Scale bars represent 100 μm. Data are shown as the mean ± S.D. Figure S3. Effect of soluble IgSF11-Fc on osteoclast differentiation. Low-cell-density cultures of wild-type BMMs were treated with the indicated doses of soluble IgSF11-Fc or Control IgG and cultured with M-CSF + RANKL for three days. On day three, cells were fixed and stained with TRAP (upper). TRAP^+^ multinucleated cells (three nuclei or more per cell) were counted and the frequency of TRAP^+^ multinucleated cells is shown (bottom). Scale bars represent 100 μm. Data are shown as the mean ± S.D. **** *p* < 0.0001.
