# Supplementary material for: CD44 Can Compensate for IgSF11 Deficiency by Associating with the Scaffold Protein PSD-95 during Osteoclast Differentiation
Source: Int J Mol Sci. 2020 Apr 10;21(7):2646. doi: 10.3390/ijms21072646 (PMC7177690; doi:10.3390/ijms21072646)
Supplement: Supplementary file 1 [file ijms-21-02646-s001.pdf]

Supplementary

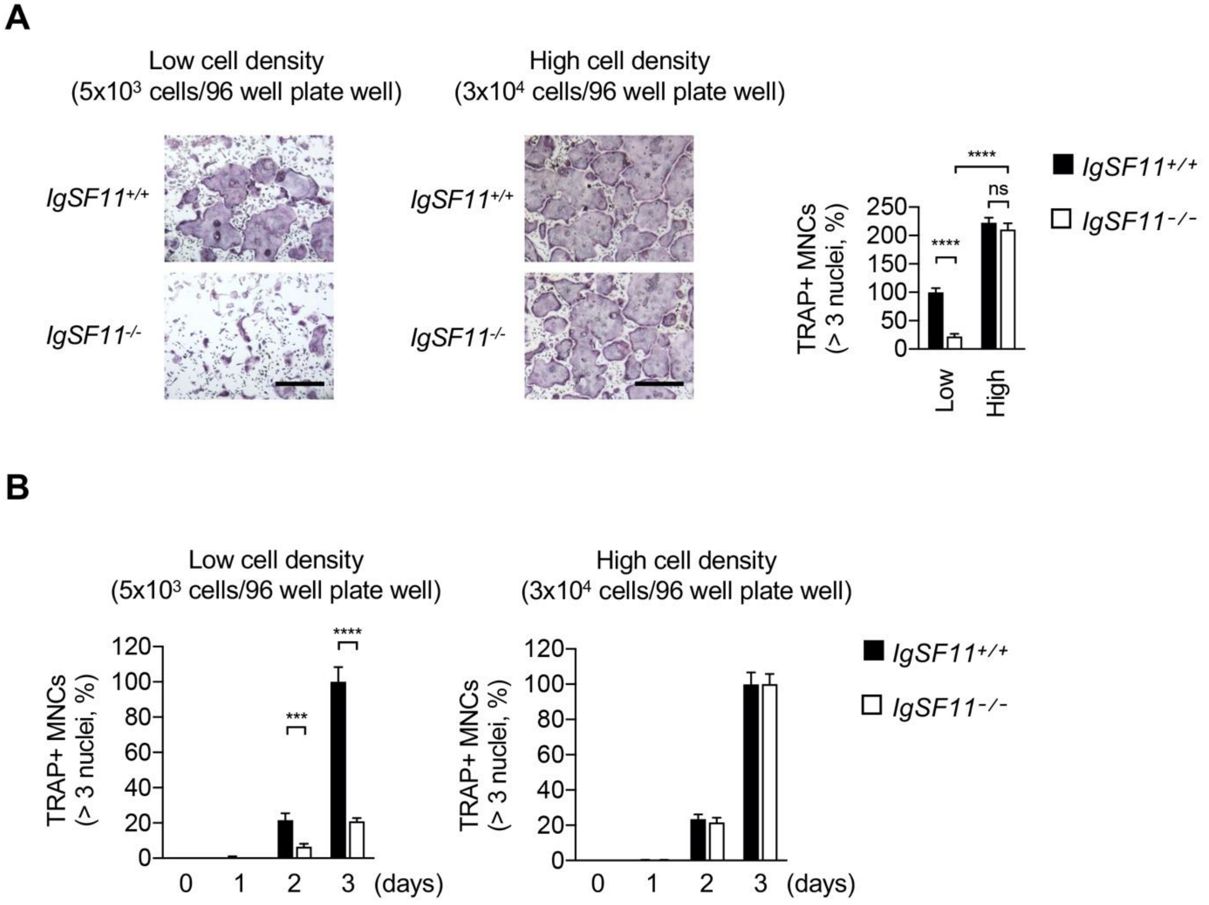

Figure S1. Increased cell density rescues impaired in vitro IgSF11-deficient osteoclast differentiation. (**A**) $IgSF11^{+/+}$ and $IgSF11^{-/-}$ BMMs were cultured at low cell density (5×10^3 cells/96 well plate well) or high cell density (3×10^4 cells/96 well plate well) with M-CSF + RANKL for three days to induce osteoclast differentiation. On day 3, cells were fixed and stained with TRAP (left). $TRAP^+$ multinucleated cells (3 nuclei or more per cell) were counted and the frequency of $TRAP^+$ multinucleated cells is shown (right). Scale bars represent 100 μm. (**B**) $IgSF11^{+/+}$ and $IgSF11^{-/-}$ BMMs were cultured at low cell density (5×10^3 cells/96 well plate well) or high cell density (3×10^4 cells/96 well plate well) with M-CSF + RANKL for three days to induce osteoclast differentiation. Cells were fixed day by day (from Day 0 to Day 3), and stained with TRAP. $TRAP^+$ multinucleated cells (3 nuclei or more per cell) were counted and the frequency of $TRAP^+$ multinucleated cells is shown. Data are shown as the mean ± S.D. ***$p < 0.001$, ****$p < 0.0001$, ns; not significant.

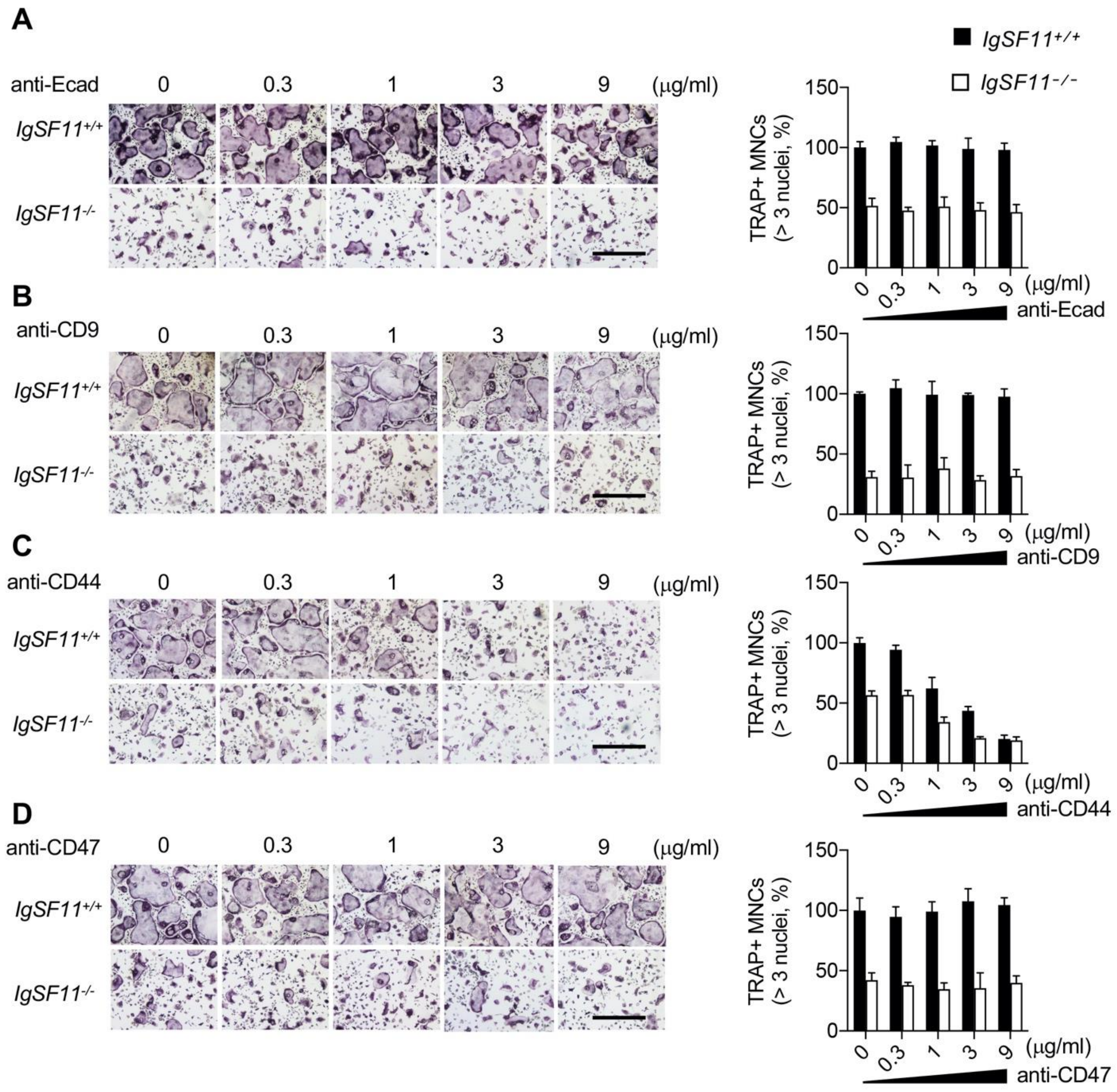

Figure S2. Effect of blocking antibodies against osteoclast differentiation/multinucleation-related molecules. Low cell density cultures of $IgSF11^{+/+}$ and $IgSF11^{-/-}$ BMMs were treated with the indicated doses of soluble antibodies (**A**) anti-E-cadherin, (**B**) anti-CD9, (**C**) anti-CD44, and (**D**) anti-CD47, and cultured with M-CSF + RANKL for three days to induce osteoclast differentiation. On day 3, cells were fixed and stained with TRAP (left). $TRAP^{+}$ multinucleated cells (3 nuclei or more per cell) were counted and the frequency of $TRAP^{+}$ multinucleated cells is shown (right). Scale bars represent 100 μm. Data are shown as the mean ± S.D.

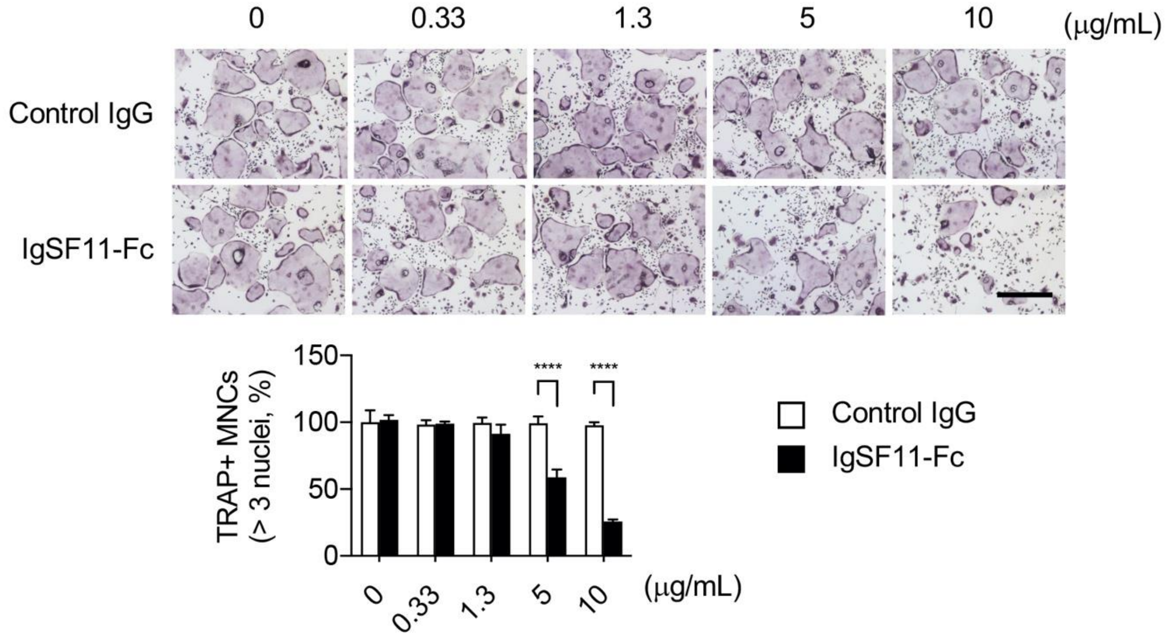

Figure S3. Effect of soluble IgSF11-Fc on osteoclast differentiation. Low cell density cultures of wild-type BMMs were treated with the indicated doses of soluble IgSF11-Fc or Control IgG, and cultured with M-CSF + RANKL for three days. On day 3, cells were fixed and stained with TRAP (upper). TRAP$^+$ multinucleated cells (3 nuclei or more per cell) were counted and the frequency of TRAP$^+$ multinucleated cells is shown (bottom). Scale bars represent 100 μm. Data are shown as the mean ± S.D. ****$p < 0.0001$.
